# Discussion of Students' E-book Reading Intention With the Integration of Theory of Planned Behavior and Technology Acceptance Model

**DOI:** 10.3389/fpsyg.2021.752188

**Published:** 2021-09-20

**Authors:** Yu-Zhou Luo, Yue-Ming Xiao, Yu-Yang Ma, Chao Li

**Affiliations:** Business School, Guilin University of Technology, Guilin, China

**Keywords:** theory of planned behavior, technology acceptance model, e-book, reading intention, behavioral intention

## Abstract

The emergence of e-books with the characteristics of easy access and reading any time anywhere is a subject of debate in academia. Topics include the use of e-books in libraries, their use in support teaching, new possibilities for reading activities, potential uses for library archives, and the motivation and intention of e-book users. Students at Guilin University of Technology participated in a survey. Of the 300 copies of the questionnaire distributed, 263 valid copies were returned, a retrieval rate of 88%. The research results show that (1) Usability and reading need are the key factors in e-book usage. Usability refers to convenient keyword searches, portability, and any time reading. E-books are considered to make searching and reading large amounts of data easier. (2) E-books are not restricted to time and space so that the overall reading quantity is increasing. Readers become accustomed to reading e-books, and the quality of their digital reading is gradually enhanced. (3) Students should complete e-book use courses offered by libraries to enhance their familiarity with e-books and their use of e-book software, thereby enhancing postgraduate student readers' e-book information literacy. The results of the research prompt suggestions to enhance the promotion of reading and e-book information to encourage student readers' e-book reading intention.

## Introduction

In the rapidly developing information era, the value of e-books gradually became apparent. The portability of e-books has been an attraction ever since their first appearance. In the field of library and information science, e-books were much discussed. Some argued that e-books should not only be used in the library but should also enhance the services that libraries provide and should be used to support teaching. Susantini et al. (2021) mentioned that it was remarked that e-books can be a new approach to reading and can change how and when people use libraries. As digital publishing expanded, libraries began to acquire significant amounts of digital products allowing users to search digital documents in a convenient and suitable manner. Almost not a country or region in the world could escape from the pandemic of Novel Coronavirus (COVID-19). The pandemic results in major changes in human life to change life and learning styles. Mass or national school closure is preceded in many countries or regions this year in order to reduce the spread of COVID-19 (Viner et al., 2020). To cope with the pandemic, the approach of “Learning never stops” allows students continuing the learning with the minimal impact. Nevertheless, in face of the menacing pandemic, most teachers passively adopt distance courses without advance warning or enough time for preparing courses (Almekhlafi, 2021). Although distance education presents various advantages and is limited to time and location (Karakoç Öztürk, 2021), the practice of distance education is the maximal transformation. A lot of countries in the world, and even schools, temporarily practice or promote distance education. It would have the frontline teachers and students face some problems or difficulties. Current academic discussions on temporary promotion of distance learning are limited, while the use of e-books is inevitable for the promotion of distance education. The rise in digital publishing led to a situation where there are more e-books than printed books (Erkayhan and Ulke, 2017). This fact is challenging for libraries, especially university libraries, where a gradual transition occurred from paper-based collections to digital resources. Users of university libraries tend to have specific needs, and building a suitable collection can be difficult. Although libraries have started to value the development of their digital collections, they lack information on readers' acceptance, requirements, usage time, and reading habits. They are unsure if readers will change their reading habits and their requirements for printed books after e-books have been introduced into libraries. This is an area worthy of discussion when libraries are compiling their collections. Therefore, the integration of the Theory of Planned Behavior (TPB) and the Technology Acceptance Model (TAM) was used in this research to investigate students' reading intention toward e-books. Understanding how readers use the collections in the libraries and assessing how their expectations are being met might improve students' reading literacy of printed books and e-books. New insights could help to promote students' reading services.

## Literature Review

### Digital Reading Behavior

In their survey of digital reading behavior, Huang and Chang (2019) found that frequent e-book reading locations included rooms at home, especially the living room, offices, schools, and on transport to work. In different situations, the most common types of e-book reading were: “business and finance,” “literature and fiction,” “tourism and sport,” “lifestyle and hobbies,” and “fashion and entertainment.” While at home, readers mainly read e-books on tablets, and they use smartphones while commuting. Regarding reading habits, 60% of respondents read 1–4 e-books a month, and about 50% of respondents read e-books for an average of 16–30 min each time. In the survey, more than 70% of the respondents were satisfied with their e-book reading experience. Leong et al. (2019) discovered that most readers read e-books on tablets, read at home, and read every week and that reading methods depend on the type of books. In terms of the effect of e-books on readers, readers' knowledge of e-books was related to the reading experience. Readers favored the convenience of e-books, but they were used to reading paper books. Ease of use of software and hardware and readers' reading habits were the key factors in reading intention. Reading e-books changed reading habits, such as searching for books, type of reading material, time spent reading, note-taking habits, frequency of repeated reading, and book sharing behavior. Readers' problems with e-books included installation, searching, and reading, with searching as the biggest problem. Readers' needs for e-books resulted in bigger collections and longer recommendation book lists. The need arose for reading software to improve searching and personalization.

### Application of Model

Kaushik and Rahman (2017) revealed that the use of the TAM to predict users' behavioral intention in using new technologies and their real-use behavior was supported by a significant body of empirical research. However, two factors, the social factor and the controlling factor, which were proven to significantly affect users' real-use behavior with new technologies, were not included in the model. These two factors were the key variables in the TPB. Wei et al. (2017) integrated the TAM and the TPB, including a subjective norm and perceived behavioral control in the TAM, proposing a combined TAM and TPB (C-TAM-TPB), and they conducted empirical research on students' use behavior of computing resource centers.

The empirical results of the study by Lee and Cranage (2018) revealed that the C-TAM-TPB, integrating the TAM and the TPB, presented a high level of fit in explaining users' use behavior with new technologies. Moreover, the group analysis of users with different use experiences revealed that C-TAM-TPB presented a good fit for users with or without experience. Therefore, in this study, TPB and TAM are integrated to discuss students' e-book reading intention.

### Research Hypothesis

Lee (2017) stated that Davis included the belief—attitude—intention—behavior relation in the theory of reasoned action in the TAM and particularly emphasized the importance of “perceived usefulness” and “perceived ease of use.” Davis considered that information technology with higher ease of use in situations with comparable tasks could assist an individual in completing more tasks in the same time, further enhancing individual work performance. In this case, the perceived ease of use could reinforce the perceived usefulness to the individual of information technology. Stouthuysen et al. (2018) mentioned that Davis also considered that users might have a negative attitude toward specific information technology but would still be willing to use the technology when its use is perceived to enhance personal work performance. Ko (2017) indicated that perceived usefulness could indirectly affect the use intention of information technology through attitude and could directly affect the behavioral intention of users. Furthermore, in addition to perceived usefulness and perceived ease of use as major factors in the attitudes of users toward information technology, attitudes would further affect the behavioral intention of users, thereby determining the acceptance and use behavior of information technology.

The following hypotheses are therefore established in this study.

H1: Perceived ease of use presents positive and direct effects on perceived usefulness.
H2: Perceived ease of use shows positive and direct effects on attitude.
H3: Perceived usefulness reveals positive and direct effects on attitude.
H4: Perceived usefulness shows positive and direct effects on behavioral intention.
H5: Attitude shows positive and direct effects on behavioral intention.
H6: Subjective norm reveals positive and direct effects on behavioral intention.
H7: Perceived behavioral control shows positive and direct effects on behavioral intention.
H8: Perceived behavioral control presents positive and direct effects on behavior.
H9: Behavioral intention reveals positive and direct effects on behavior.

## Methodology

### Conceptual Structure

**Figure 1:**
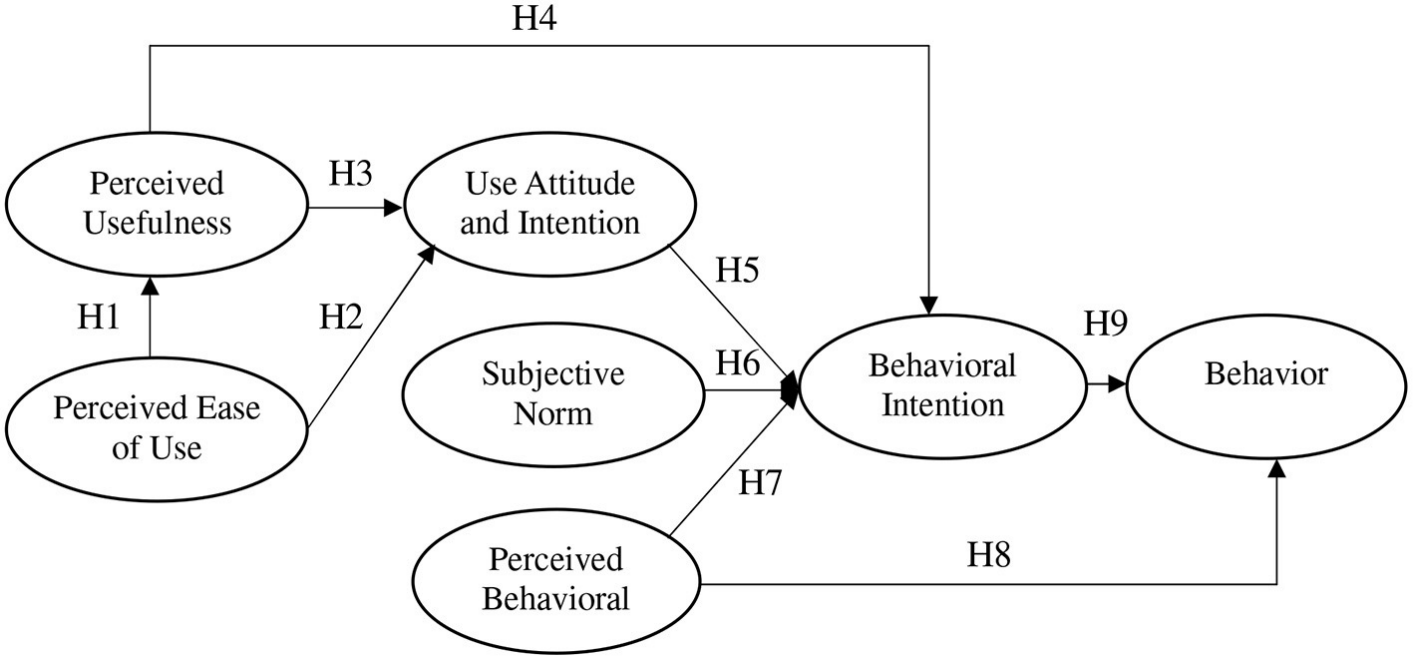
Conceptual structure.

The integration of the TAM and the TPB is used to construct the model in this study, as shown in Figure 1.

### Research Subject and Analysis Method

Five hundred copies of a questionnaire were distributed to university students in Guilin, China. A total of 433 valid copies were returned, a retrieval rate of 87%. AMOS software was used as the data analysis tool to evaluate students' e-book reading intention.

## Result

Confirmatory Factor Analysis (CFA) results show that convergent validity in the observation model could observe the suggested reliability value of individual observed variables, construct reliability (CR), and average variances extracted (AVE), where the reliability of individual observed variables is suggested as higher than 0.5. The factor loadings of various observed variables in this study are higher than the suggested value. A CR value of higher than 0.6 is preferable, but some researchers suggest that it should be higher than 0.5. The estimation result of the model shows that the CR is higher than 0.5. The average variance extracted should be higher than 0.5. The average variance extracted of dimensions in this study is higher than 0.5, conforming to the suggested value.

The estimation results of the structural equation are shown in Table 1. First, the suggested standards for X^2^/df, RMSEA, GFI, AGFI, RMR, and NFI are ≦ 5, ≦ 0.08, ≧ 0.9, ≧ 0.9, ≦ 0.05, and ≧ 0.9, respectively. The values in this study appear as X^2^/df = 3.134 ≦ 5, RMSEA = 0.034 ≦ 0.08, GFI = 0.968 ≧ 0.9, AGFI = 0.925 ≧ 0.9, RMR = 0.03 ≦ 0.05, and NFI = 0.942 ≧ 0.9, revealing good overall fit. As a result, the estimation results of the structural equation (Table 1) show that all parameters achieve the significant standards (*p* < 0.05).

**Table 1:** Structural equations model result.

**Parameter/evaluation standard**	**Coefficient**	***T***
Perceived ease of use → perceived usefulness	0.134	2.422^**^
Perceived ease of use → attitude	0.177	3.288^**^
Perceived usefulness → attitude	0.183	2.735^**^
Perceived usefulness → behavioral intention	0.196	3.626^***^
Attitude → behavioral intention	0.167	4.257^***^
Subjective norm → behavioral intention	0.171	4.962^***^
Perceived behavioral control → behavioral intention	0.206	5.583^***^
Perceived behavioral control → behavior	0.157	1.927^**^
Behavioral intention → behavior	0.214	3.641^**^
χ^2^/degree of freedom ≦5	3.134	
Root mean square error of approximation (RMSEA) ≦0.08	0.034	
Goodness-of-fit index (GFI) ≧0.9	0.968	
Adjusted goodness-of-fit index (AGFI) ≧0.9	0.925	
Root mean square residual (RMR) ≦0.05	0.03	
Normed fit index (NFI) ≦0.9	0.942	

** *,*

*** *Stands for the significance under the significant standard of 5%*.

From the above estimation results of the structural equations, the following seven research hypotheses in this study are supported: H1: Perceived ease of use presents positive and direct effects on perceived usefulness; H2: Perceived ease of use shows positive and direct effects on attitude; H3: Perceived usefulness reveals positive and direct effects on attitude; H4: Perceived usefulness shows positive and direct effects on behavioral intention; H5: Attitude shows positive and direct effects on behavioral intention; H6: Subjective norm reveals positive and direct effects on behavioral intention; and H7: Perceived behavioral control shows positive and direct effects on behavioral intention.

## Discussion

Most e-books are free and easy to access. Therefore, students tend to read quickly and to immediately reject unsuitable e-books and to search for the next one. In the reading process of acquisition and searching, e-books usually show an abstract on the interface indicating the content and facilitating the elimination of unsuitable books or articles. Printed books do not often show abstracts, and the content is only indicated by the cover. Students appear to have a higher acceptance of e-books and to have definite reading goals, thereby increasing reading frequency. Furthermore, students generally appreciate the convenience of keyword searches of e-books, discovering new reading strategies involving integration and explanation. Students tend to read printed books for comprehension and to use keywords to directly search for solutions in e-books. It seems that an increasing number of students are engaging in digital reading for academic purposes.

## Conclusion

The research results reveal students' high demands for reading e-books. The convenience of not being restricted to time and space is popular among students when considering the importance of e-books. The results show that students are not familiar with e-book resources and how to use them, and that digital reading can be limited by software and hardware issues. In addition, note-taking can be challenging because of the unfamiliar interface. Therefore, students should participate actively in the e-book use education courses offered by libraries to increase their understanding of e-books and to improve their ability to use e-book reading software to enhance their e-book information literacy. Students encounter many difficulties in the digital reading experience, including the limitations of software and hardware, the challenges involved in underlining and making notes, and the unfriendly user interface. Students expressed the view that unstable software or hardware might hinder their e-book reading. E-book firms could improve the stability of software and hardware, thereby enhancing students' use satisfaction. It is therefore suggested that e-book firms should regularly survey readers' digital reading experiences and improve the e-book service to increase students' e-book use intention.

## Data Availability Statement

The original contributions presented in the study are included in the article/supplementary material, further inquiries can be directed to the corresponding author.

## Ethics Statement

The present study was conducted in accordance with the recommendations of the Ethics Committee of the Guilin University of Technology, with written informed consent being obtained from all the participants. The research protocol was approved by the ethical committee of the Guilin University of Technology.

## Author Contributions

Y-ZL performed the initial analyses and wrote the manuscript. Y-MX was responsible for the methodology, software, and validation. Y-YM and CL assisted in the data collection and data analysis. All authors revised and approved the submitted version of the manuscript.

## Funding

This research was supported by the Guangxi Zhuang Autonomous Region: 100 Plan on the Introduction of High-level Overseas Talents for Colleges and Universities in Guangxi (YM 20181204).

## Conflict of Interest

The authors declare that the research was conducted in the absence of any commercial or financial relationships that could be construed as a potential conflict of interest.

## Publisher's Note

All claims expressed in this article are solely those of the authors and do not necessarily represent those of their affiliated organizations, or those of the publisher, the editors and the reviewers. Any product that may be evaluated in this article, or claim that may be made by its manufacturer, is not guaranteed or endorsed by the publisher.
